# Constructing a novel gene signature derived from oxidative stress specific subtypes for predicting survival in stomach adenocarcinoma

**DOI:** 10.3389/fimmu.2022.964919

**Published:** 2022-08-18

**Authors:** Renlong Zhou, Naixiong Peng, Wei Li

**Affiliations:** ^1^ Department of Blood Transfusion, Shenzhen Longhua District Central Hospital, Shenzhen, China; ^2^ Department of Urology, Shenzhen Longhua District Central Hospital, Shenzhen, China

**Keywords:** Oxidative stress, stomach adenocarcinoma, immune cell infiltration, risk signature, immunotherapy

## Abstract

Oxidative stress (OS) response is crucial in oncogenesis and progression of tumor. But the potential prognostic importance of OS-related genes (OSRGs) in stomach adenocarcinoma (STAD) lacked comprehensive study. STAD clinical information and transcriptome data were retrieved from the Gene Expression Omnibus and The Cancer Genome Atlas databases. The prognostic OSRGs were filtered *via* the univariate Cox analysis and OSRG-based molecular subtypes of STAD were developed using consensus clustering. Weighted gene co-expression network analysis (WGCNA) was subsequently conducted to filter molecular subtype-associated gene modules. The prognosis-related genes were screened *via* univariate and least absolute shrinkage and selection operator Cox regression analysis were used to construct a prognostic risk signature. Finally, a decision tree model and nomogram were developed by integrating risk signature and clinicopathological characteristics to analyze individual STAD patient’s survival. Four OSRG-based molecular subtypes with significant diversity were developed based on 36 prognostic OSRGs for STAD, and an OSRGs-based subtype-specific risk signature with eight genes for prognostic prediction of STAD was built. Survival analysis revealed a strong prognostic performance of the risk signature exhibited in predicting STAD survival. There were significant differences in mutation patterns, chemotherapy sensitivity, clinicopathological characteristics, response to immunotherapy, biological functions, immune microenvironment, immune cell infiltration among different molecular subtypes and risk groups. The risk score and age were verified as independent risk factors for STAD, and a nomogram integrating risk score and age was established, which showed superior predictive performance for STAD prognosis. We developed an OSRG-based molecular subtype and identified a novel risk signature for prognosis prediction, providing a useful tool to facilitate individual treatment for patients with STAD.

## Introduction

Stomach adenocarcinoma (STAD) is the most common histological type (95%) of stomach tumors and on of the top three causes leading to cancer mortality (1). STAD is highly metastatic and the majority of STAD patients occur lymph node metastasis when diagnosed (1). Currently, for STAD patients the most effective treatment is the integration of radical surgery and chemotherapy at the early stages. But still, most of STAD patients are in advanced stage when diagnosed, resulting in a loss of optimal chances for taking surgery (2). In addition, about 40% of STAD patients will progress to metastasis and recurrence after surgery. Consequently, despite great advances in therapeutic techniques, the survival and prognosis of advanced STAD remain unsatisfactory (3). Hence, to develop novel prognostic and diagnostic biomarkers for STAD is an urgent task.

Oxidative stress (OS) is caused by an imbalance of synthesis between oxidants and antioxidants and is accompanied by reactive oxygen species (ROS) accumulation, participating in carcinogenesis and cancer development (4). ROS overproduction can lead to a wide spectrum of DNA lesions and genotoxic damage, eventually resulting in tumorigenesis (5). OS-related genes (OSRGs) play crucial roles in countering OS and have been reported to profoundly affect a variety of processes, including proliferation, differentiation, and angiogenesis (6–8). Studies have reported that OSRGs are predictive of cancers such as gliomas (9), bladder cancer (10), and prostate cancer (11). The potential role of OSRGs in the tumorigenesis and progression of STAD has been gradually demonstrated by growing evidence (12–15). Nonetheless, limited knowledge is available about the molecular pattern of OSRGs in STAD and the link of OSRGs with clinical features, immune landscape, and response to immunotherapy, and drug sensitivity.

To comprehensively understand the role of oxidative stress in STAD development as well as its potential in STAD treatment, we explored the expression patterns of OSRGs and constructed OSRG-related molecular subtypes. We assessed the crosstalk between OSRGs and immune microenvironment, responsiveness to immunotherapy or chemotherapy based on OSRG-related molecular subtypes. Furthermore, we built a prognostic risk signature from the hub gene set associated with OSRG-based subtypes and established a novel nomogram for STAD prognostic prediction. Furthermore, the link of immune landscape, responsiveness to immunotherapy, and drug sensitivity, clinical characteristics, with the risk signature was studied. To the best of our knowledge, this was the first STAD classification based on OSRG signatures and elucidated how OSRG signatures were intrinsically linked to the response to chemotherapy and immunotherapy and immune microenvironment in detail. It may provide new insights into the pathophysiology of STAD, facilitating more tailored treatments and improving the outcomes for STAD patients.

## Materials and methods

### Data collection and preparation

The transcriptome data and clinical information of STAD patients were obtained from TCGA and GEO databases. The patients without complete follow-up, status information, or clinical information of shorter than 30 days were excluded. Totally 337 TCGA patients were retained as a training cohort, and 291 patients of the GSE15459 (182 patients) and GSE26901 (109 patients) cohorts were utilized for validation. The median value was taken as the gene expression value when a gene ID corresponded to multiple probes in GEO datasets or when multiple gene symbols existed in the TCGA cohort. The human OSRGs were obtained from the “GOBP_RESPONSE_TO_OXIDATIVE_STRESS” pathway in the MSigDB database.

### Consensus clustering

The prognostically correlated OSRGs were found *via* the univariate Cox regression analysis, to explore the correlation among mRNA expression of these prognostic OSRGs, and Spearman correlation analysis was conducted. On the basis of prognostic OSRGs, the consensus clustering analysis in “ConsensusClusterPlus” R package was carried out to generate molecular subtypes (16). “KM” algorithm and “1-Pearson correlation” was selected as distance for conducting 500 runs of bootstraps, with each run containing 80% of samples. We chose Cluster number k from 2 to 10. The optimal number of cluster was determined by cumulative distribution curve (CDF) and consensus matrix. To compare the prognosis among the subtypes, Kaplan-Meier (K-M) analysis was executed. And the expression of prognosis-related OSRGs among the subtypes was heatmapped and clustered. We performed single-sample gene set enrichment analyses (ssGSEA) to acquire the OS ssGSEA scores of molecular subtypes in “GSVA” R.

### WGCNA

WGCNA in the “WGCNA” R package was performed on TCGA cohort for the identification of the key module associated with the molecular subtypes and the underlying molecular connections (17). The average linkage method and Pearson’s correlation matrices were adopted for all paired genes, and using the absolute value of the correlation of transcriptional data, a co-expression similarity matrix was constructed. Next, weighted adjacency matrix based on the co-expression similarity matrix with appropriate power of β was calculated. Then the weighted adjacency matrix was converted into topological overlap matrix (TOM). Dynamic tree cut method was used for identifying modules, which were clustered then. The correlations between gene module and OSRG-based subtypes were calculated to identify the hub module, which was selected for further analysis. We also performed Spearman correlation analysis to investigate gene significance and its correlation with modules. And the “clusterprofiler” R package was applied to perform enrichment analysis (18).

### Prognostic signature construction and efficacy evaluation

The hub module associated with the molecular subtype was identified using WGCNA analysis. Then, to screen the prognosis-related genes in the hub module, univariate Cox regression analysis was adopted, and LASSO regression analysis was used to build a risk score using the following formula: risk score = ∑β_i_× Exp_i_. Where the β_i_ is the coefficient and Exp_i_ is gens’ normalized level of expression. STAD patients in the TCGA and GEO cohorts were divided into two risk groups (low and high) by the median risk score value. To explore the prognostic significance of the risk score in STAD, K-M survival analysis was conducted with log-rank test, and the risk signature was evaluated with receiver operating characteristic (ROC) curve in the survivalROC and timeROC packages (19).

### Gene set enrichment analyses (GSEA)

GSEA on TCGA data was carried out to investigate the biological functions of those selected genes to infer their impact with GSEA (version 4.0.3). Statistical significance was P-value < 0.05 and the false discovery rate (FDR) < 0.05. In the Hallmark database all candidate gene sets were used in the GSEA analysis. In addition, the gene sets of ferroptosis and autophagy are obtained from the “WP_FERROPTOSIS” and “GOBP_REGULATION_OF_AUTOPHAGY” pathways in the MSigDB database, respectively, and the inflammatory- and angiogenesis-related gene sets are derived from literature (20, 21).

### Assessment of immune landscape

The proportions of 22 immune cell subtypes in the TCGA cohort were evaluated by CIBERSORT algorithm, a tool for assessing immune cell infiltration (22), and the stromal and immune scores were calculated using ESTIMATE algorithm to further explore tumor microenvironment (TME).

### Prediction of responsiveness to immunotherapy and chemotherapy

The TIDE algorithm is a tool for the prediction of responsiveness to immune checkpoint inhibitors, and it was applied to evaluate the responsiveness to immune checkpoint inhibitors in the TCGA cohort using the transcriptome data (23). The TIDE algorithm calculates three scores that limit T cell infiltration, including the M2 subtype of tumor-associated macrophages (TAM) score, myeloid-derived suppressor cells (MDSCs) score, tumor-associated fibroblasts (CAF) score. Meanwhile, the dysfunction score of tumor-infiltrating cytotoxic T lymphocytes (CTLs) and the rejection score of CTLs by immunosuppressive factors were also assessed. Additionally, the “pRRophetic” in the R calculated estimated biochemical half maximal inhibitory concentration (IC50) and forecast the sensitivity of samples to four chemotherapeutic drugs in the TCGA cohort (24).

### Immune checkpoint and single-sample GSEA (ssGSEA)

Expressions of TCGA immune checkpoint genes were analyzed and compared according to the molecular subtype and risk signature. We performed ssGSEA analysis in “GSVA” R package to evaluate the activities of interested pathways in the molecular subtypes and different risk groups (25).

### Mutation analysis

The TMB was calculated for each sample as somatic mutation numbers (nonsynonymous mutations) per megabase in the coding region of tumor genome, and 20 TCGA genes or from different risk groups with the most frequent mutation were illustrated and analyzed using the “GenVisR” R package (26). K-M analysis with log-rank test and GSEA analysis was performed for the Mutant and wild-type (WT) groups.

### Statistical analysis

The R software (v3.6.3) performed all statistical analyses. The correlation matrices were conducted using Pearson or Spearman correlation. Wilcoxon test was conducted for two-group comparisons. Survival differences were compared using K–M curves with a Log-rank test. Statistical significance was P-value < 0.05.

## Results

### The mutation patterns and transcriptome characteristics of OSRGs in STAD

**Figure 1:**
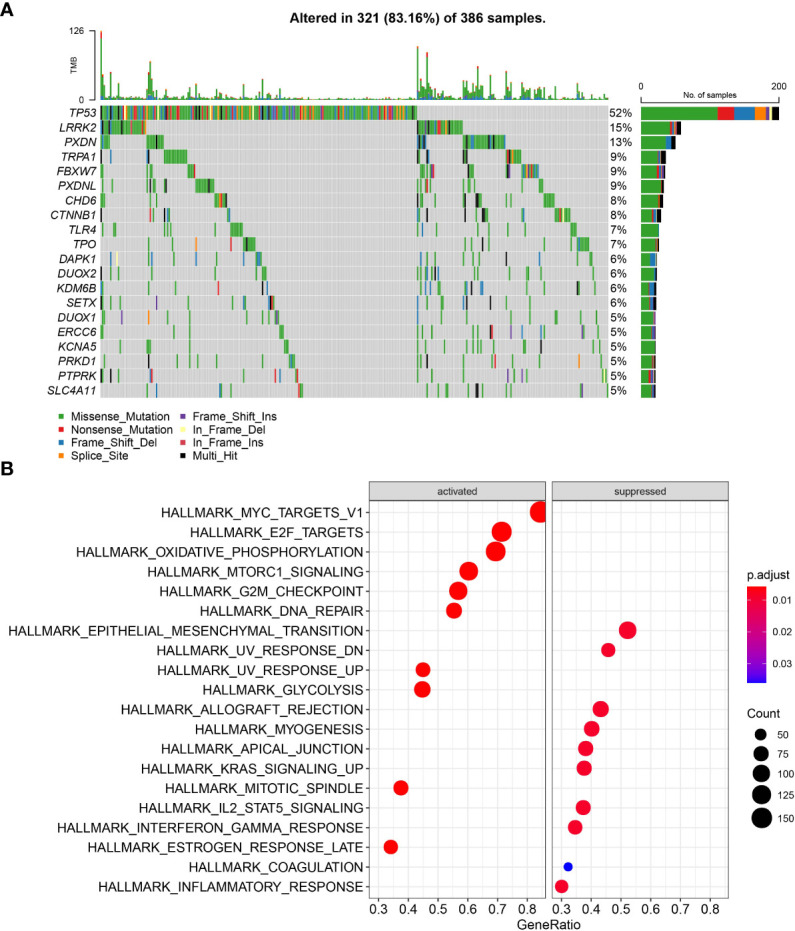
The mutation patterns and transcriptome characteristics of OSRGs in STAD. **(A)** Mutation profile of OSRGs in patients with STAD; **(B)** GSVA analysis between the mutant group and the WT group.

A total of 436 OSRGs were identified in this study and the mutations of OSRGs in STAD were analyzed. There are 386 (88.3%) patients had mutations in OSRGs, among which the top20 OSRGs were shown in **Figure 1A**. Among them, the mutation frequency of TP53 gene was the highest, followed by LRRK2 and PXDN. TCGA patients were then categorized into the Mutant group and the WT group according to the mutations in OSRGs. We compared the biological functions between the mutant group and the WTgroup by Gene Set Variation Analysis (GSVA) enrichment analysis. Tumor-related gene setssuch as ALLMARK_E2F_TARGETS, HALLMARK_G2M_CHECKPOINT, HALLMARK_MYC_TARGETS_V1, HALLMARK_DNA_REPAIR, were enriched in the mutant group, while in WT group immune-related pathways were significantly enriched (**Figure 1B**). This indicated that the mutation of OSRGs may lead to functional changes and affect the survival and prognosis of patients with STAD.

### Consensus clustering on OSRGs

**Figure 2:**
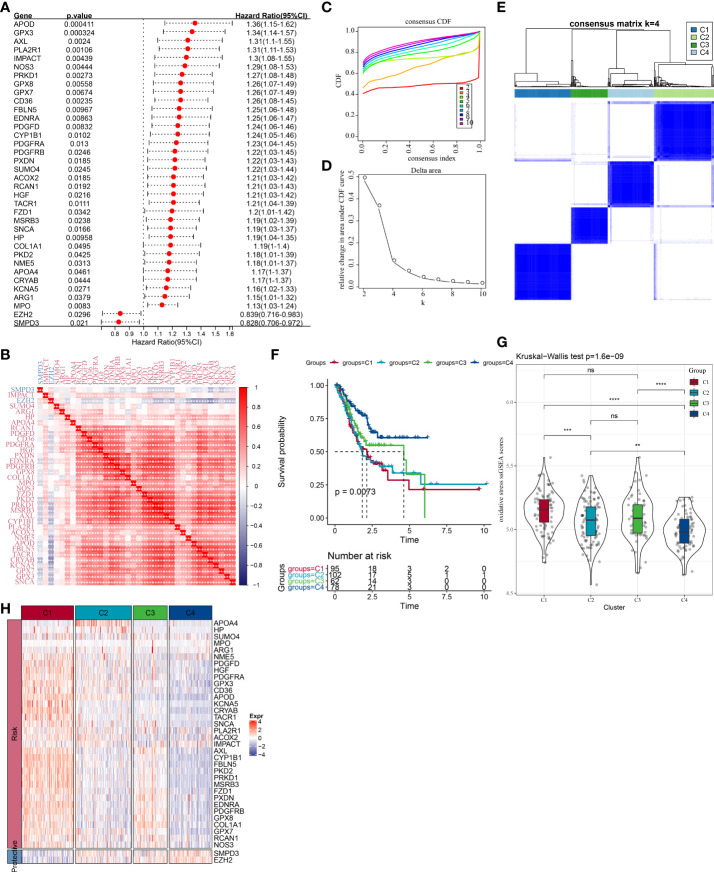
Molecular clustering based on OSRGs in the TCGA cohort. **(A)** Forest plot of OSRGs that correlated with prognosis; **(B)** Correlation matric of prognosis-related OSRGs; **(C)** Consensus cumulative distribution function (CDF) diagram when different k values. **(D)** Delta area plot for relative change in the area under CDF curve for k compared to k-1. **(E)** Consensus matrix when number of groups (k) = 3. In the consensus matrix, white meant that samples were impossibly clustered together, and dark blue meant that samples were always clustered together. Both rows and columns of the matrix represented samples. **(F)** Kaplan-Meier curves for overall survival of four molecular subtypes (number of C1 = 95, C2 = 102, C3 = 62, and C4 = 78). The survival probabilities were compared with log-rank test. **(G)** Comparison of OS ssGSEA score among the C1, C2, C3, and C4 subtypes in the TCGA cohort. **(H)** Clustering analysis of the expression of the 36 prognostic OSRGs. ns, no significance. *P <  0.05, **P <  0.01, ***P <  0.001, ****P <  0.0001.

To understand the OSRGs expression patterns, we identified 36 prognosis-related OSRGs in the TCGA cohort *via* univariate Cox regression analysis (**Figure 2A**). Here, 34 “risk” OSRGs contribute to poor prognosis while the rest 2 “protective” OSRGs were related to a better outcome. The correlation matric of the expression of the 36 OSRGs was illustrated in **Figure 2B**, which showed significant correlations between different OSRGs. According to the expression profiles of the 36 OSRGs, STAD patients in TCGA were classified into four subtypes (C4, C3, C2, C1) (**Figures 2C–E**). Survival analysis revealed significant prognostic differences among four subtypes (p=0.0073), as shown in **Figure 2F**. The C1 subtype exhibited the poorest prognosis while the C4 subtype showed the best outcome. We calculated the “OS ssGSEA scores” of each TCGA patient having STAD, and found that the C4 subtype had the lowest “OS ssGSEA scores” whereas the C1 subtype has the highest one (**Figure 2G**). Additionally, we compared the expression of 36 OSRGs in different molecular subtypes (**Figure 2H**) and found that the “risk” gene was high-expressed in C1, while the “protective” gene was high-expressed in C4.

### Clinicopathological characteristics and mutation patterns in the different molecular subtypes

**Figure 3:**
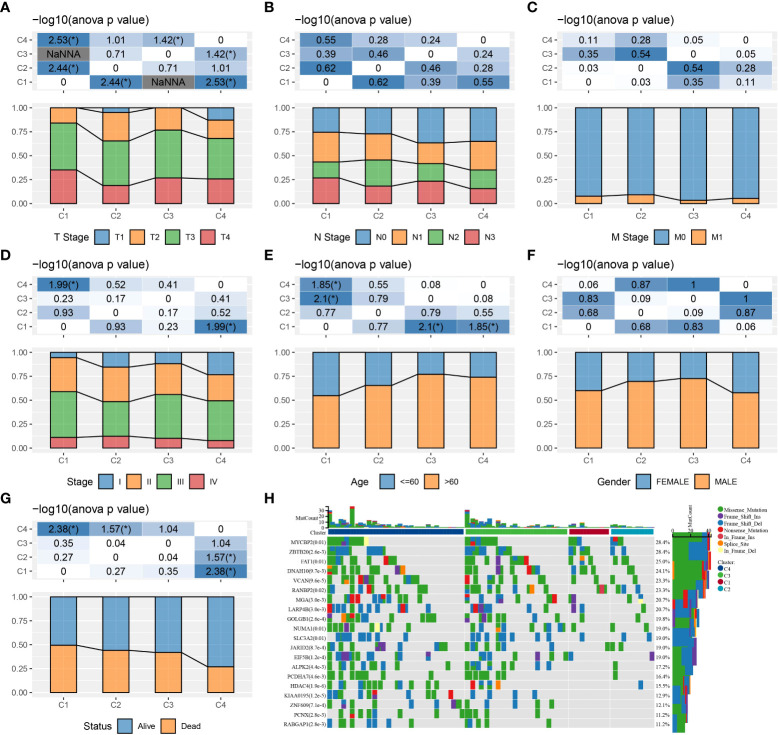
The clinicopathological and mutation characteristics of molecular subtypes in the TCGA cohort. **(A–G)** Comparison of T stage, N stage, M stage, clinical stage, age, gender, and outcome status among different molecular subtypes. **(H)** Waterfall plots of frequently mutated genes in four molecular subtypes. *P < 0.05.

We compared the differences in clinicopathological characteristics and mutation patterns according to the molecular subtypes. As shown in **Figure 3**, our data revealed that significant differences occurred in the T stage, stage, age, and status between different molecular subtypes. Specifically, the C1 subtype exhibited an advanced grade and a higher mortality rate. Meanwhile, among different molecular subtypes, OSRGs with high SNP frequency were different. In the waterfall map, 20 genes, which had the highest mutation rate in the C1, C2, C3, and C4 subtypes, respectively, were displayed. In addition, four molecular subtypes also presented different genomic features including homologous recombination defects, aneuploidy, tumor mutation burden, fraction altered, number of segments (**Figure S1A**). Furthermore, we obtained the molecular subtypes of STAD from previous studies (27, 28) and compared the correlations between our defined molecular subtypes and other molecular subtypes (**Figures S1B, C**). C3 and C4 subtypes had higher proportion of MSI subtypes, consistent with a study in which cancer patients with MSI demonstrated a more favorable prognosis.

### Immune landscape and responsiveness to immunotherapy and chemotherapy in the different molecular subtypes

**Figure 4:**
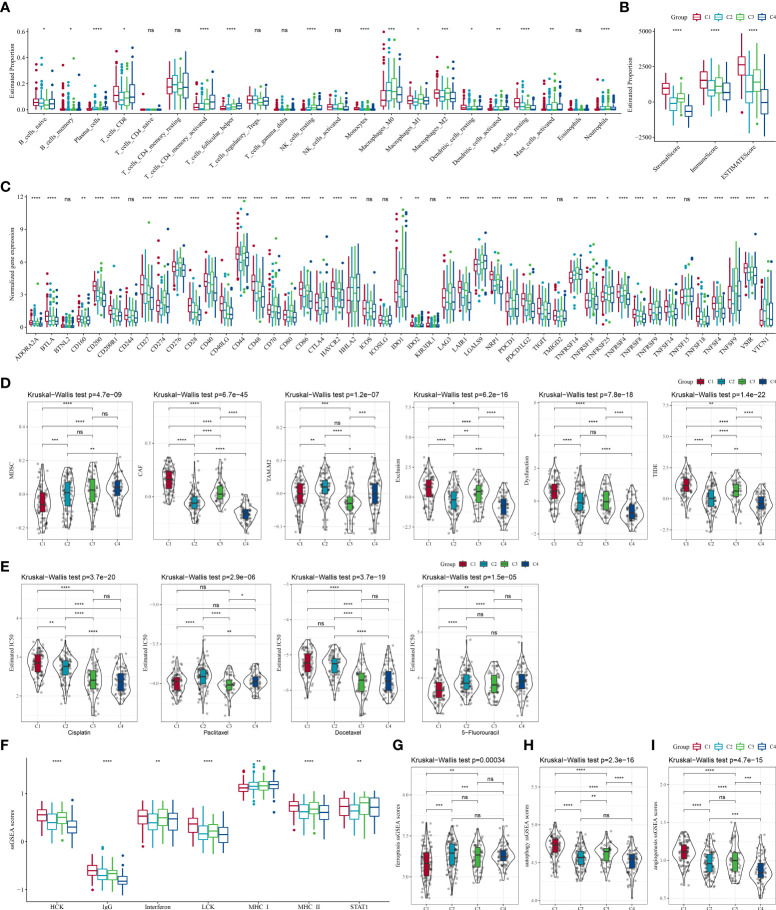
The differences in the immune microenvironment and responsiveness to immunotherapy and chemotherapy among molecular subtypes in the TCGA cohort. **(A)** Estimated proportions of 22 immune cells among the four subtypes; **(B)** Comparison of the stromal score, immune score, and estimate score in the four subtypes; **(C)** The expression of immune checkpoints genes in the four subtypes; **(D)** Differences in TIDE analysis results between different subtypes; **(E)** The box plots of the estimated IC_50_ for cisplatin, paclitaxel, docetaxel and 5-fluorouracil in different subtypes; **(F)** The ssGSEA scores of seven inflammation-related gene in different subtypes; **(G–I)** The ssGSEA scores of ferroptosis, autophagy, angiogenesis-related gene sets in different subtypes. ns, no significance. *P <  0.05, **P <  0.01, ***P <  0.001, ****P <  0.0001.

The infiltration of immune cells of TCGA patients were assessed. CIBERSORT analysis revealed that a vast majority of immune cells showed a different infiltration level among the four subtypes (**Figure 4A**). Similar results were also shown in TIMER analysis (**Figure S2A**). The immune score in other subtypes were noticeably lower than the C1 subtype, indicating an elevated immune infiltration in the C1 subtype (**Figure 4B**). Significant differences in the expression level of most immune checkpoint genes among the four subtypes were detected (P < 0.05, **Figure 4C**). Meanwhile, we found that the C4 subtype presented a lower CAF score, T cell exclusion score, T cell dysfunction score, TIDE score, and a higher MDSC score than the other three subtypes (**Figure 4D**). High TIDE score pointed to a greater probability of immune escape and a lower probability to benefit from immunotherapy. Thus, our data implied that STAD patients of C4 could benefit from immunotherapy. Drug sensitivity analysis showed that the four molecular subtypes exerted significantly different responsiveness to chemotherapy (**Figure 4E**). It demonstrated that the C1 subtype was more reactive to 5-fluorouracil than the other subtypes, while the C4 subtype showed more responsive to cisplatin and docetaxel than the rest subtypes. Furthermore, differences were detected to be significant among the four subtypes in the ssGSEA score of ferroptosis, autophagy, angiogenesis, and seven inflammation-related genes (**Figures 4F–I**).

### Gene modules associated with molecular subtype derived from WGCNA

**Figure 5:**
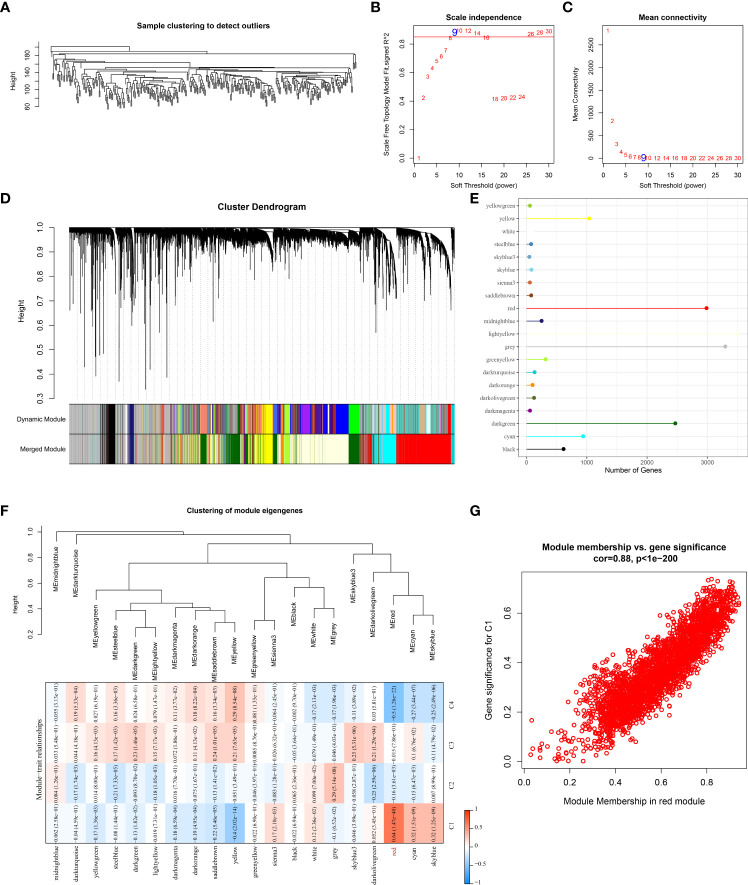
Identification of gene modules that associated with molecular subtyps by WGCNA analysis in the TCGA cohort. **(A)** Cluster analysis of samples of STAD in the TCGA cohort; **(B)** Analysis of the scale-free fit index for various soft-thresholding powers **(β)**; **(C)** Analysis of the mean connectivity for various soft-thresholding powers. **(D)** Hierarchical clustering dendrogram of co-expressed genes in modules in STAD; **(E)** The number of genes in each gene module; **(F)** Correlation between the module eigenvectors of each module and molecular subtypes; **(G)** Scatter diagram for module membership vs. gene significance for C1 subtype in the red module.

We performed WGCNA analysis for the development of co-expression networks and identification of gene modules related to molecular subtypes. The results of hierarchical clustering of the TCGA samples was in **Figure 5A**. When pointing at values 9, the research of an adequate soft-threshold verifying converged toward scale-free topology (**Figures 5B, C**). Firstly, dynamic tree cut algorithm (Dynamic Module) helped distinguish gene modules that were then merged (Merged Module) with the following criterion: r > 0.75, height = 0.3, deepSplit = 2, minModuleSize = 30, as shown in **Figure 5D**. Finally, we identified 20 gene modules in STAD and gene number in each module was illustrated in **Figure 5E**. We compared the correlations between the modules and molecular subtypes, and the data demonstrated that the red module was negatively related to the C4 subtype (r = -0.5, p = 1.26e-22) and significantly positively associated with the C1 subtype (r = 0.64, p = 1.47e-40) (**Figure 5F**). The module membership in the red module was positively linked with the gene significance (r = 0.88, p < 1e-5) in the C1 subtype, as shown in **Figure 5G**. The biological functions of genes in the red module were enriched and illustrated in **Figure S3**.

### Construction and validation of an OSRGs-based prognostic signature

**Figure 6:**
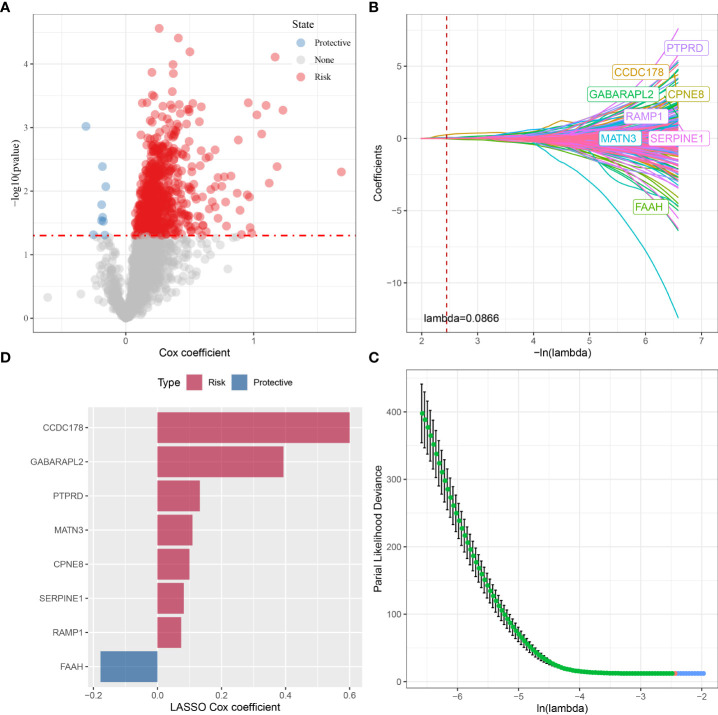
The identification of prognostic OSRGs for risk signature in the TCGA cohort. **(A)** The identification of prognostic OSRGs from the red module *via* univariate COX regression analysis. **(B)** The LASSO analysis was used to identify the prognostic variables and develop the predictive models. **(C)** Plots of the produced coefficient distributions for the logarithmic (lambda) series for parameter selection (lambda). **(D)** The LASSO Cox coefficients for each OSRGs in the risk signature.

To build an OSRG-based prognostic signature, we firstly screened 908 prognostic OSRGs (p < 0.05) from the red module *via* univariate COX regression analysis (**Figure 6A**), which consisted of 899 “risk” genes and 9 “protective” genes. According to the results of the LASSO analysis, eight prognostic OSRGs, including CCDC178, GABARAPL2, PTPRD, MATN3, CPNE8, SERPINE1, RAMP1, and FAAH, were further screened out based on the optimal lambda value (0.0866) (**Figures 6B–D**).

**Figure 7:**
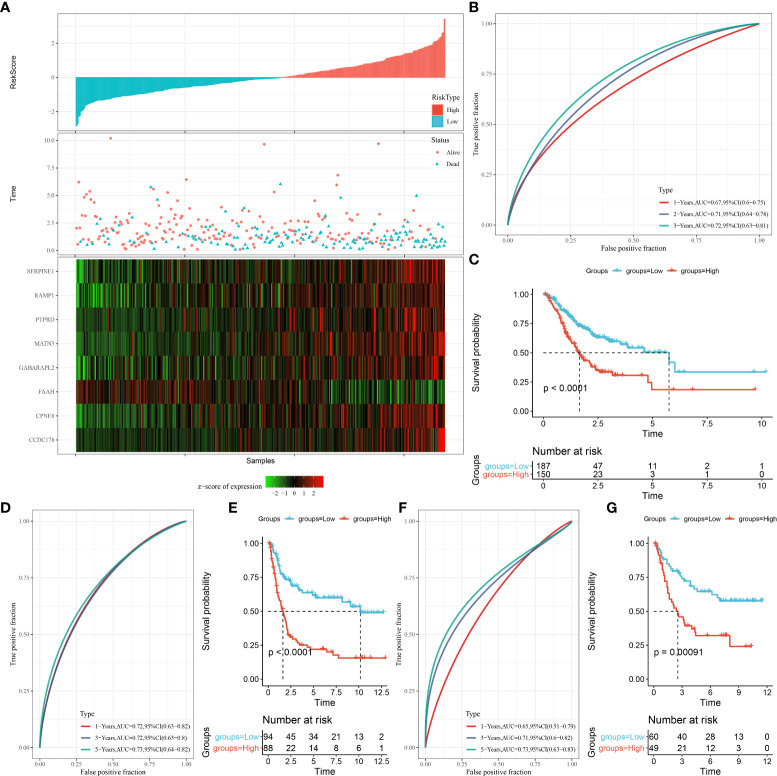
The construction of OSRG-based prognostic signature in the TCGA cohort. **(A)** The distribution of patient longevity status and risk score, and the expression profiles of nine aging genes in high and low risk group in TGGA cohort. **(B, D, F)** represent the receiver operating characteristic curves for forecasting overall survival in the TCGA cohort, GSE21257, and GSE16091 cohort, respectively. **(C, E, G)** represent the survival curves for patients with high risk score and low risk score in TCGA cohort.

Each STAD sample (in the TCGA cohort) were categorized into two risk groups (low, high) by the median risk score (**Figure 7A**). High-risk STAD patients showed an favorable prognostic outcome compared with the low-risk group (**Figure 7B**, p < 0.0001). As shown in **Figure 7C**, AUCs value for 1-, 2-, and 3-year overall survival of STAD patients in the TCGA cohort were 0.67, 0.71, and 0.72, respectively. This was further validated in the GSE21257 and GSE16091 cohorts (**Figures 7D–G**). These data suggested that, the risk model performed well and was robust in predicting prognosis of STAD patients.

Moreover, we compared our prognostic model with other four models from previous studies. Here, this study applied the same methodology for calculating the risk score of other models in TCGA cohort, and classified samples into two risk groups based on z-score = 0. ROC analysis and survival analysis were performed on the four models. Compared to these models, our model showed the most favorable performance of AUC (0.67, 0.71, and 0.72 for 1, 3, and 5-year prognosis respectively, see **Figures S4A–D**). Furthermore, we calculated C-index of the five models, and our model showed the highest C-index value (0.65), suggesting the best performance of our model in predicting prognosis (**Figure S4E**).

### Clinicopathological characteristics in different risk groups

The correlations between the clinicopathological characteristics and the risk signature were analyzed by stratified analyses. The results showed great differences in the risk score according to the stratification by M stage, gender, N stage, status, Stage, age, molecular subtypes, T stage (**Figure S5A**). Younger STAD patients (≤ 60 years) showed higher risk scores as compared with patients over 60 years old. The risk score was lower in the T1 stage than in the other stages as stratified by T stage grade. Patients with metastatic STAD had higher risk scores than those without metastasis. STAD patients in early stage had the lowest risk scores than other advanced clinical stages. The risk scores of deceased samples were noticeably higher than those who were alive. Meanwhile, the risk score of C1 was significantly higher, while that of C4 was lower. In the gender and N stage, no significant differences were observed. Correspondingly, difference comparison in clinicopathological characteristics between the two risk groups (**Figure S5B**). The results revealed significant differences in molecular subtypes, age, T stage, outcome status between the two risk groups. Furthermore, K-M subgroup analysis showed that the OS of STAD between the two risk groups was noticeably different based on stratification by age, gender, and clinical stage (**Figure S5C**).

### Immune infiltration and biological functions in the different risk groups

**Figure 8:**
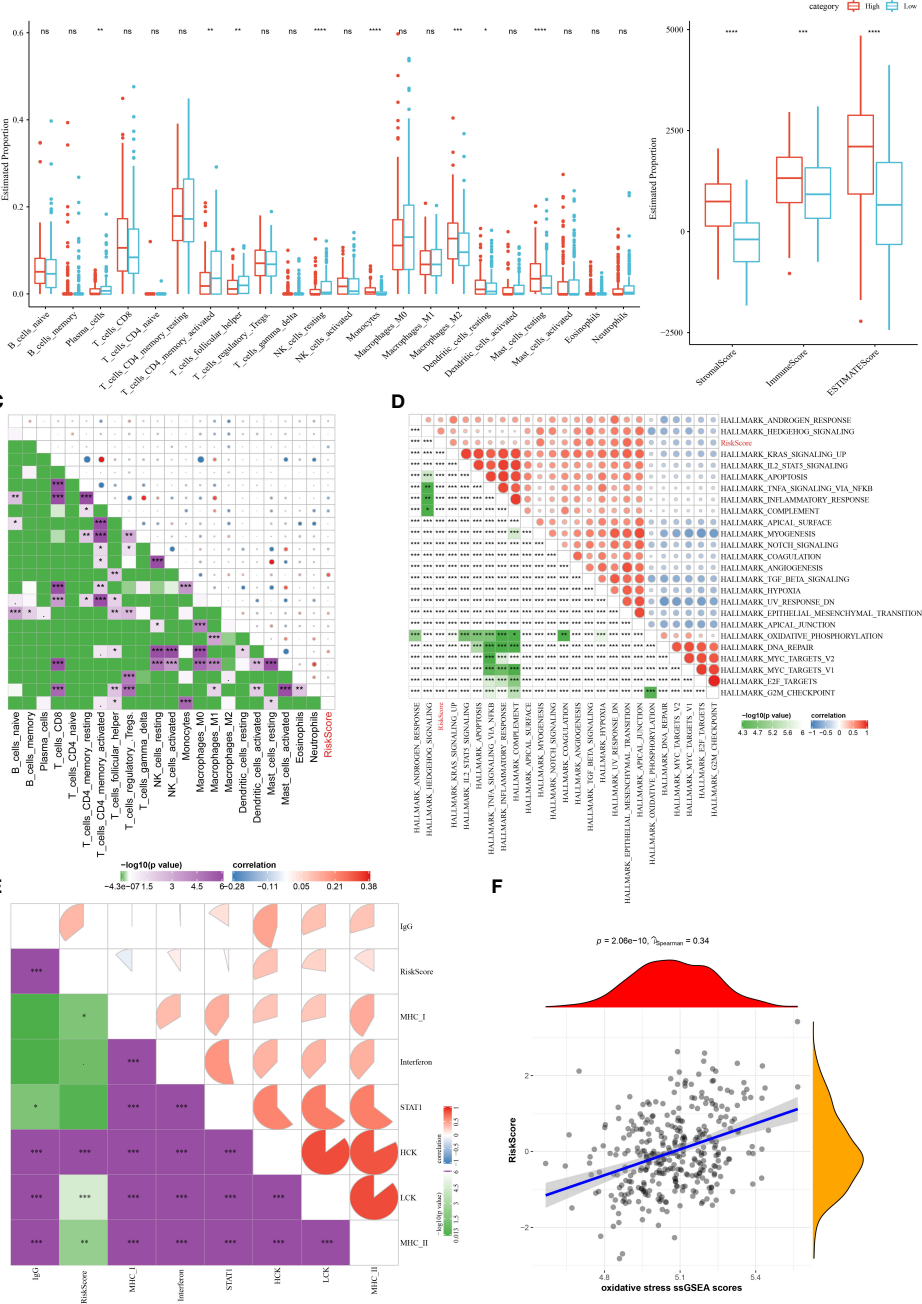
The associations between immune infiltration and biological functions in different risk group in the TCGA cohort. **(A)** The proportion of 22 immune cells between different risk groups; **(B)** Comparison of the stromal score, immune score, and ESTIMATE score between the high- and low-risk groups. **(C–F)** Correlation analysis of the risk score and 22 immune cells, biological functions, inflammation-related genes, as well as the OS ssGSEA scores. ns, no significance. *P <  0.05, **P <  0.01, ***P <  0.001, ****P <  0.0001.

To clarify the differences in patients’ immune microenvironment of the two risk groups, the relative abundance of 22 immune cells was compared. There are eight immune cell subtypes that showed significant differences in infiltration levels in STAD. In low-risk group, the infiltration levels of the resting NK cells, activated CD4 memory T cell, plasma cells, follicular helper T cells were higher than high-risk patients, while the infiltration level of M2 macrophages, resting dendritic cells, resting mast cells, monocytes in the high-risk group were significantly higher than the low-risk group (**Figure 8A**). Similar data were also shown in TIMER analysis (**Figure S2B**). In comparison with low-risk samples, those show of a high risk had a higher immune score, ESTIMATE score, stromal score (**Figure 8B**). The correlation matric found significantly positive correlations between the risk score and the immune infiltration of monocytes and resting mast cells. Meanwhile, the risk score was negatively related to the follicular helper T cells and immune infiltration of activated CD4 memory T cells (**Figure 8C**). Furthermore, we performed ssGSEA analysis to reveal the connections between biological functions and the risk score. Significant correlations were observed between the risk score and biological functions as well as inflammation-related genes, as shown in **Figures 8D, E**. In addition, we noticed that the OS ssGSEA score was positively associated with the risk score (r = 0.34, p = 2.06e-10) (**Figure 8F**).

### Risk signature predicts responsiveness to immunotherapy and chemotherapy

The majority of immune checkpoint genes in different risk groups were differently expressed (**Figure S6A**). Furthermore, the TIDE analysis revealed a significantly higher CAF score, T cell exclusion score, T cell dysfunction score, and TIDE score, and a lower MDSC score in the high-risk group (**Figure S6B**). These data indicated that STAD patients with low-risk scores are more likely to benefit from immunotherapy. Additionally, the risk score was revealed to be positively related to the CAF, TIDE,T cell dysfunction, and T cell exclusion scores (**Figure S6C**). In the TCGA cohort, patients with a low risk were more sensitive to cisplatin and docetaxel treatments than high-risk ones (**Figure S6D**). These data suggested that the OSRG-based signature showed a close association with the responsiveness to immunotherapy and chemotherapy.

### Improvement of the prognostic model *via* integrating risk signature and clinical features

**Figure 9:**
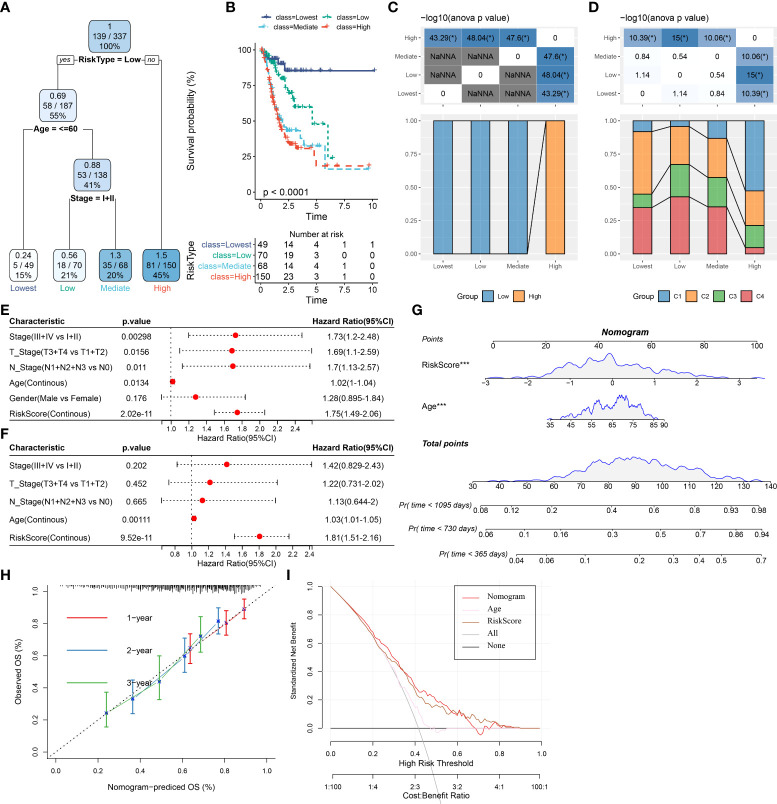
Improvement of prognostic model *via* integrating risk signature and clinical features in the TCGA cohort. **(A)** Patients with full-scale annotations including risk score, metastatic, gender and age were used to build a survival decision tree to optimize risk stratification. **(B)** Comparison of overall survival of the four subgroup obtained from the decision tree model. **(C)** Correlations between the four subgroup and the risk signature. **(D)** Correlations between the four subgroup and molecular subtypes. **(E)** Univariate and **(F)** multivariate Cox analysis of risk score and clinicopathological characteristics in the TCGA cohort. **(G)** A nomogram combining risk signature and age was generated in the TCGA cohort. **(H)** Comparison of the calibration curve for 1-, 2-, 3- year overall survival of nomogram. **(I)** Decision curves for the clinical net benefit of each model in comparison to all or none strategies. The x-axis indicated the threshold probability, and the y-axis indicated the net clinical benefit. *P <  0.05.

To develop a better prognostic model for STAD, we constructed a decision tree model and multivariate Cox regression model *via* combining the OSRG-based risk signature and other clinical parameters. Our data showed that the patients can be stratified into four distinct groups (Lowest, Low, Mediate, High) using a decision tree on only risk score, age, and clinical stage (**Figure 9A**). From survival curves, the overall survival of STAD among the four defined subgroups showed significant differences (**Figure 9B**). Patients in the “Lowest”, “Low”, and “Mediate” subgroups belong to the OSRG-based low-risk group, while the “High” subgroup belongs to the OSRG-based high-risk group (**Figure 9C**). In addition, C1 and C2 subtypes occupy more than the C3 subtype in the “Highest” group (**Figure 9D**). The risk score was seen as the most significant independent prognostic factor of STAD (HR = 1.81, 95%CI: 1.51 - 2.16, *P* = 9.52e-11), followed by age (HR = 1.03, 95%CI: 1.01 - 1.05, *P* = 0.00111) (**Figures 9E, F**). Therefore, a nomogram was then generated using the risk score and age for the prediction of STAD prognosis (**Figure 9G**). The nomogram can effectively forecast the actual survival outcomes, as shown by calibration curve (**Figure 9H**). Furthermore, the DCA curve demonstrated that the nomogram and risk signature had better prognostic capacity than other clinicopathological features, as shown in **Figure 9I**.

## Discussion

Although great advances in diagnostic and surgical techniques in recent years, the overall survival of patients with advanced STAD remains poor. There is an urgent need to develop novel prognostic biomarkers of STAD and reveal the mechanisms of tumor progression. Much evidence has linked OS to tumorigenesis and progression, and OSRGs, which regulate OS processes, have been shown to have great potential as biomarkers and therapeutic targets. Although a recent study reported the value of OSRGs in STAD prognosis (29), the relationship between OSRGs characteristics and STAD clinicopathological features, responsiveness to chemotherapy and immunotherapy, immune microenvironment remains unclear. In our study, we first identified 36 OSRGs that are associated with STAD prognosis and the functions of these genes in STAD have been gradually elucidated in recent years. For instance, the elevated expression of the platelet-derived growth factor D (PDGFD) was proved to be an indicator for a poor prognostic outcome of patients with gastric cancer (GC) (13), and the PDGFD-related immune-gene signature was regarded as a moderator to guide immunotherapy programs. Glutathione peroxidase 3 (GPX3) has been confirmed to prevent migration and invasion in GC *via* NF-κB/Wnt5a/JNK signaling (15). Meanwhile, the hypermethylation of GPX3 in GC forecasts a shorter tumor recurrence time in patients aged > 60 (14). A recent study demonstrated that CD36 can mediate palmitate acid-induced metastasis of GC (12). However, the functions and underlying mechanisms of prognosis-related OSRGs in STAD need to be further exhaustively investigated. According to the best of our knowledge, were are the first to construct molecular subtypes based on these OSRGs for STAD and reveal their associations with clinicopathological features, immune microenvironment, immunotherapy response and chemosensitivity in STAD patients.

STAD is a typical heterogeneous malignancy with different subtypes and clinical behaviors (30). The OSRG-based subtypes of STAD also exhibited distinct diversity with regard to the clinicopathological features, immune microenvironment, and immunotherapy response and chemosensitivity. ROS was reported to be involved in the regulation of immune checkpoint genes. An enhanced generation of ROS was able to promote PD-L1 expression in tumor cells (31), and we also observed differential expression of immune checkpoint genes among OSRG-based molecular subtypes. This implicates possible differences in immunotherapy response of these subtypes and the potential of these subtypes to aid in the development of an individual’s immunotherapy strategies. Subsequent TIDE analysis further revealed differential immunotherapy responses between subtypes. The OSRG-based subtype was noticed to be correlated with two subtypes (microsatellite instability and Epsterin-Barr virus subtypes), which have been confirmed to be more likely to benefit from immune checkpoint blockade (32–34). The chemosensitivity is thought to be associated with OS in STAD. The cisplatin resistance in GC cells could be potentiated by mitochondrial dysfunction by targeting a ROS-activated pathway (35), and piperlongumine could enhance the antitumor efficacy of oxaliplatin *via* inducing ROS in GC cells (36). Our results demonstrated that the OSRG-based subtypes showed different chemotherapy sensitivity, implying the potential role of OSRGs in drug resistance to STAD.

In this study, WGCNA was performed to select the hub gene module that related to the OSRG-based subtypes, and a novel risk signature was developed from these hub genes *via* lasso regression analysis. This OSRG-based subtype-specific gene signature was identified as an independent prognostic factor with a robust prognostic value for STAD, and subsequent analyses further confirmed that this risk signature has a huge advantage in the prognostic prediction for patients with STAD. The nomogram model integrating the risk signature and age exhibited better inspection efficiency than that of other clinicopathological features. In addition, our data revealed significant differences in the clinical features, immune infiltration, and responsiveness to immunotherapy and chemotherapy between two risk groups. The risk signature could be applied to the prognosis treatment and prediction of STAD patients.

Herein, the OSRG-based subtype-specific risk signature enrolled eight genes, which is broadly implicated in tumor progression and initiation. The coiled-coil domain-containing protein 178 (CCDC178) has been found to be mutated in hepatocellular carcinoma (HCC) (37) and GC cells (38), and it was found to promote HCC metastasis by mediating anoikis (39). GABA type A receptor-associated protein-like 2 (GABARAPL2) was involved in protein transport and membrane fusion events and has been proved to be related to autophagy (40). Protein tyrosine phosphatase receptor delta (PTPRD) was frequently decreased in GC and was correlated with worse a higher risk of distant metastasis, overall survival, advanced stage (41). Previous studies found that Martrilin-3 (MATN3) was abnormally expressed and associated with the prognosis of GC (42, 43), and recent studies also confirmed the diagnostic and prognostic value of MATN3 for GC from comprehensive data mining (44, 45). Copine 8 (CPNE8) was preferentially expressed in ovarian CCC compared to HGSC. However, few studies were conducted to explore the effects of CPNE8 in tumorigenesis. Serpin family E member 1 (SERPINE1) encodes plasminogen activator inhibitor 1 (PAI-1), which regulates extracellular matrix (ECM) remodeling through a direct inhibition of plasminogen activators (46). SERPINE1 was involved in the TME remodeling and immune infiltration and its overexpression was revealed to be associated with poor patient outcome in various cancers (47, 48). The receptor activity-modifying protein 1 (RAMP1) could promote tumorigenesis in prostate cancer (49). Fatty acid amide hydrolase (FAAH) hydrolyzes the endocannabinoid anandamide and other N-acylethanolamines, which have been reported with anti-cancer effects (50).

However, our study had some limitations. The current research was conducted using retrospective data from public databases. Therefore, it should be validated in more prospective and multi-center STAD cohorts in the future. Secondly, our results still need *in vivo* or *in vitro* experiments to validate our mechanism analysis. Therefore, further studies are required to explore the underlying mechanisms of the signature in the development of STAD.

## Conclusion

In conclusion, this study developed four molecular subtypes with distinct diversity based on 36 prognostic OSRGs and constructed a novel prognostic risk signature of STAD. We further elucidated the immune landscape, biological functions, drug sensitivity, and immunotherapy response according to the molecular subtypes and risk groups. The risk signature and age were identified as independent risk factors of STAD, and a nomogram combining the novel risk signature and age was developed. It may serve as a clinical tool for making personalized therapeutic treatment and forecasting prognosis for patients with STAD.

## Data availability statement

The datasets presented in this study can be found in online repositories. The names of the repository/repositories and accession number(s) can be found in the article/**Supplementary Material**.

## Author contributions

RZ and WL designed the study. RZ and NP contributed to the literature research, analyzed and interpreted the data. RZ wrote the initial draft of the manuscript. WL reviewed and edited the manuscript. All authors contributed to the article and approved the submitted version.

## Conflict of interest

The authors declare that the research was conducted in the absence of any commercial or financial relationships that could be construed as a potential conflict of interest.

## Publisher’s note

All claims expressed in this article are solely those of the authors and do not necessarily represent those of their affiliated organizations, or those of the publisher, the editors and the reviewers. Any product that may be evaluated in this article, or claim that may be made by its manufacturer, is not guaranteed or endorsed by the publisher.

## References

[1] Ajani JA Lee J Sano T Janjigian YY Fan D Song S. Gastric adenocarcinoma. Nat Rev Dis Primers (2017) 3:17036. doi: 10.1038/nrdp.2017.36 28569272

[2] Song Z Wu Y Yang J Yang D Fang X. Progress in the treatment of advanced gastric cancer. Tumour Biol (2017) 39(7):1010428317714626. doi: 10.1177/1010428317714626 28671042

[3] Coutzac C Pernot S Chaput N Zaanan A. Immunotherapy in advanced gastric cancer, is it the future? Crit Rev Oncol Hematol (2019) 133:25–32. doi: 10.1016/j.critrevonc.2018.10.007 30661655

[4] Hayes JD Dinkova-Kostova AT Tew KD. Oxidative stress in cancer. Cancer Cell (2020) 38(2):167–97. doi: 10.1016/j.ccell.2020.06.001 PMC7439808 32649885

[5] Moloney JN Cotter TG. ROS signalling in the biology of cancer. Semin Cell Dev Biol (2018) 80:50–64. doi: 10.1016/j.semcdb.2017.05.023 28587975

[6] Golstein P Kroemer G. Cell death by necrosis: towards a molecular definition. Trends Biochem Sci (2007) 32(1):37–43. doi: 10.1016/j.tibs.2006.11.001 17141506

[7] Shimazaki T Noro N Hagikura K Matsumoto T Yoshida-Noro C. Quantitative analysis of factors regulating angiogenesis for stem cell therapy. Biol (Basel) (2021) 10(11):1212. doi: 10.3390/biology10111212 PMC8614798 34827205

[8] Rus A Robles-Fernandez I Martinez-Gonzalez LJ Carmona R Alvarez-Cubero MJ. Influence of oxidative stress-related genes on susceptibility to fibromyalgia. Nurs Res (2021) 70(1):44–50. doi: 10.1097/NNR.0000000000000480 32991532

[9] Lu D Yang N Wang S Liu W Zhang D Wang J. Identifying the predictive role of oxidative stress genes in the prognosis of glioma patients. Med Sci Monit (2021) 27:e934161. doi: 10.12659/MSM.934161 34836934 PMC8634738

[10] Zhang M Du G Li Z Li D Li W Li H. An oxidative stress-related genes signature for predicting survival in bladder cancer: based on tcga database and bioinformatics. Int J Gen Med (2022) 15:2645–67. doi: 10.2147/IJGM.S348945 PMC8922338 35300137

[11] Zhang Z Jiang D Wang C Garzotto M Kopp R Wilmot B. Polymorphisms in oxidative stress pathway genes and prostate cancer risk. Cancer Causes Control (2019) 30(12):1365–75. doi: 10.1007/s10552-019-01242-7 31667711

[12] Pan J Fan Z Wang Z Dai Q Xiang Z Yuan F. CD36 mediates palmitate acid-induced metastasis of gastric cancer *via* AKT/GSK-3β/β-catenin pathway. J Exp Clin Cancer Res (2019) 38(1):52. doi: 10.1186/s13046-019-1049-7 30717785 PMC6360779

[13] Yang Y Kang W Yuan Y Duan C Chen W Yu C. Circ-0007707/miR-429/PDGFD pathway regulates the progression of gastric cancer by modulating the immune-gene signature. J Oncol 2022 (2022) 2022:2214686. doi: 10.1155/2022/2214686 PMC9061023 35509844

[14] Zhou C Pan R Li B Huang T Zhao J Ying J. GPX3 hypermethylation in gastric cancer and its prognostic value in patients aged over 60. Future Oncol (2019) 15(11):1279–89. doi: 10.2217/fon-2018-0674 30924352

[15] Cai M Sikong Y Wang Q Zhu S Pang F Cui X. Gpx3 prevents migration and invasion in gastric cancer by targeting NFкB/Wnt5a/JNK signaling. Int J Clin Exp Pathol (2019) 12(4):1194–203. PMC6947061 31933934

[16] Wilkerson MD Hayes DN. ConsensusClusterPlus: a class discovery tool with confidence assessments and item tracking. Bioinformatics (2010) 26(12):1572–3. doi: 10.1093/bioinformatics/btq170 PMC2881355 20427518

[17] Langfelder P Horvath S. WGCNA: an r package for weighted correlation network analysis. BMC Bioinf (2008) 9:559. doi: 10.1186/1471-2105-9-559 PMC2631488 19114008

[18] Yu G Wang LG Han Y He QY. clusterProfiler: an r package for comparing biological themes among gene clusters. OMICS (2012) 16(5):284–7. doi: 10.1089/omi.2011.0118 PMC3339379 22455463

[19] Heagerty PJ Zheng Y. Survival model predictive accuracy and ROC curves. Biometrics (2005) 61(1):92–105. doi: 10.1111/j.0006-341X.2005.030814.x 15737082

[20] Liu Q Cheng R Kong X Wang Z Fang Y Wang J. Molecular and clinical characterization of pd-1 in breast cancer using large-scale transcriptome data. Front Immunol (2020) 11:558757. doi: 10.3389/fimmu.2020.558757 33329517 PMC7718028

[21] Masiero M Simões FC Han HD Snell C Peterkin T Bridges E. A core human primary tumor angiogenesis signature identifies the endothelial orphan receptor ELTD1 as a key regulator of angiogenesis. Cancer Cell (2013) 24(2):229–41. doi: 10.1016/j.ccr.2013.06.004 PMC3743050 23871637

[22] Kawada JI Takeuchi S Imai H Okumura T Horiba K Suzuki T. Immune cell infiltration landscapes in pediatric acute myocarditis analyzed by CIBERSORT. J Cardiol (2021) 77(2):174–8. doi: 10.1016/j.jjcc.2020.08.004 32891480

[23] Jiang P Gu S Pan D Fu J Sahu A Hu X. Signatures of T cell dysfunction and exclusion predict cancer immunotherapy response. Nat Med (2018) 24(10):1550–8. doi: 10.1038/s41591-018-0136-1 PMC6487502 30127393

[24] Geeleher P Cox N Huang RS. pRRophetic: an r package for prediction of clinical chemotherapeutic response from tumor gene expression levels. PloS One (2014) 9(9):e107468. doi: 10.1371/journal.pone.0107468 25229481 PMC4167990

[25] Hänzelmann S Castelo R Guinney J. GSVA: gene set variation analysis for microarray and RNA-seq data. BMC Bioinf (2013) 14:7. doi: 10.1186/1471-2105-14-7 PMC3618321 23323831

[26] Skidmore ZL Wagner AH Lesurf R Campbell KM Kunisaki J Griffith OL. GenVisR: Genomic visualizations in r. Bioinformatics (2016) 32(19):3012–4. doi: 10.1093/bioinformatics/btw325 PMC5039916 27288499

[27] Thorsson V Gibbs DL Brown SD Wolf D Bortone DS Ou Yang TH. The immune landscape of cancer. Immunity (2018) 48(4):812–30.e14. doi: 10.1016/j.immuni.2018.03.023 29628290 PMC5982584

[28] Liu Y Sethi NS Hinoue T Schneider BG Cherniack AD Sanchez-Vega F. Comparative molecular analysis of gastrointestinal adenocarcinomas. Cancer Cell (2018) 33(4):721–35.e8. doi: 10.1016/j.ccell.2018.03.010 29622466 PMC5966039

[29] Wu Z Wang L Wen Z Yao J. Integrated analysis identifies oxidative stress genes associated with progression and prognosis in gastric cancer. Sci Rep (2021) 11(1):3292. doi: 10.1038/s41598-021-82976-w 33558567 PMC7870842

[30] Hudler P. Challenges of deciphering gastric cancer heterogeneity. World J Gastroenterol (2015) 21(37):10510–27. doi: 10.3748/wjg.v21.i37.10510 PMC4588074 26457012

[31] Glorieux C Xia X He YQ Hu Y Cremer K Robert A. Regulation of PD-L1 expression in K-ras-driven cancers through ROS-mediated FGFR1 signaling. Redox Biol (2021) 38:101780. doi: 10.1016/j.redox.2020.101780 33171331 PMC7658718

[32] Goodman AM Sokol ES Frampton GM Lippman SM Kurzrock R. Microsatellite-stable tumors with high mutational burden benefit from immunotherapy. Cancer Immunol Res (2019) 7(10):1570–3. doi: 10.1158/2326-6066.CIR-19-0149 PMC6774837 31405947

[33] Duffy MJ Crown J. Biomarkers for predicting response to immunotherapy with immune checkpoint inhibitors in cancer patients. Clin Chem (2019) 65(10):1228–38. doi: 10.1373/clinchem.2019.303644 31315901

[34] Jin Z Yoon HH. The promise of PD-1 inhibitors in gastro-esophageal cancers: microsatellite instability vs. PD-L1 J Gastrointest Oncol (2016) 7(5):771–88. doi: 10.21037/jgo.2016.08.06 PMC5056248 27747091

[35] Wang SF Chen MS Chou YC Ueng YF Yin PH Yeh TS. Mitochondrial dysfunction enhances cisplatin resistance in human gastric cancer cells *via* the ROS-activated GCN2-eIF2α-ATF4-xCT pathway. Oncotarget (2016) 7(45):74132–51. doi: 10.18632/oncotarget.12356 PMC5342041 27708226

[36] Zhang P Shi L Zhang T Hong L He W Cao P. Piperlongumine potentiates the antitumor efficacy of oxaliplatin through ROS induction in gastric cancer cells. Cell Oncol (Dordr) (2019) 42(6):847–60. doi: 10.1007/s13402-019-00471-x PMC12994355 31493144

[37] Huang J Deng Q Wang Q Li KY Dai JH Li N. Exome sequencing of hepatitis b virus-associated hepatocellular carcinoma. Nat Genet (2012) 44(10):1117–21. doi: 10.1038/ng.2391 22922871

[38] Zhang J Huang JY Chen YN Yuan F Zhang H Yan FH. Whole genome and transcriptome sequencing of matched primary and peritoneal metastatic gastric carcinoma. Sci Rep (2015) 5:13750. doi: 10.1038/srep13750 26330360 PMC4557136

[39] Hu X Zhao Y Wei L Zhu B Song D Wang J. CCDC178 promotes hepatocellular carcinoma metastasis through modulation of anoikis. Oncogene (2017) 36(28):4047–59. doi: 10.1038/onc.2017.10 28319061

[40] Jacquet M Guittaut M Fraichard A Despouy G. The functions of Atg8-family proteins in autophagy and cancer: linked or unrelated? Autophagy (2021) 17(3):599–611. doi: 10.1080/15548627.2020.1749367 32255730 PMC8032235

[41] Bae WJ Ahn JM Byeon HE Kim S Lee D. PTPRD-inactivation-induced CXCL8 promotes angiogenesis and metastasis in gastric cancer and is inhibited by metformin. J Exp Clin Cancer Res (2019) 38(1):484. doi: 10.1186/s13046-019-1469-4 31805999 PMC6896474

[42] Zhang C Liang Y Ma MH Wu KZ Dai DQ. KRT15, INHBA, MATN3, and AGT are aberrantly methylated and differentially expressed in gastric cancer and associated with prognosis. Pathol Res Pract (2019) 215(5):893–9. doi: 10.1016/j.prp.2019.01.034 30718100

[43] Wu PL He YF Yao HH Hu B. Martrilin-3 (matn3) overexpression in gastric adenocarcinoma and its prognostic significance. Med Sci Monit (2018) 24:348–55. doi: 10.12659/MSM.908447 PMC5784332 29343680

[44] Li D Xu J Dong X Chen W Pan L Jiang H. Diagnostic and prognostic value of MATN3 expression in gastric carcinoma: TCGA database mining. J Gastrointest Oncol (2021) 12(4):1374–83. doi: 10.21037/jgo-21-267 PMC8421911 34532095

[45] Wang P Xiao WS Li YH Wu XP Zhu HB Tan YR. Identification of MATN3 as a novel prognostic biomarker for gastric cancer through comprehensive TCGA and GEO data mining. Dis Markers (2021) 2021:1769635. doi: 10.1155/2021/1769635 34900024 PMC8660198

[46] Andreasen PA Egelund R Petersen HH. The plasminogen activation system in tumor growth, invasion, and metastasis. Cell Mol Life Sci (2000) 57(1):25–40. doi: 10.1007/s000180050497 10949579 PMC11146824

[47] Teng F Zhang JX Chen Y Shen XD Su C Guo YJ. LncRNA NKX2-1-AS1 promotes tumor progression and angiogenesis *via* upregulation of SERPINE1 expression and activation of the VEGFR-2 signaling pathway in gastric cancer. Mol Oncol (2021) 15(4):1234–55. doi: 10.1002/1878-0261.12911 PMC8024734 33512745

[48] Sun X Cai Y Hu X Mo M Zhao C He W. Long noncoding RNA MAFG-AS1 facilitates bladder cancer tumorigenesis *via* regulation of miR-143-3p/SERPINE1 axis. Transl Cancer Res (2020) 9(11):7214–26. doi: 10.21037/tcr-20-1971 PMC8799265 35117325

[49] Logan M Anderson PD Saab ST Hameed O Abdulkadir SA. RAMP1 is a direct NKX3.1 target gene up-regulated in prostate cancer that promotes tumorigenesis. Am J Pathol (2013) 183(3):951–63. doi: 10.1016/j.ajpath.2013.05.021 PMC3763771 23867798

[50] Brunetti L Loiodice F Piemontese L Tortorella P Laghezza A. New approaches to cancer therapy: combining fatty acid amide hydrolase (faah) inhibition with peroxisome proliferator-activated receptors (ppars) activation. J Med Chem (2019) 62(24):10995–1003. doi: 10.1021/acs.jmedchem.9b00885 31407888

