# Supplementary figures and images for: Constructing a novel gene signature derived from oxidative stress specific subtypes for predicting survival in stomach adenocarcinoma
Source: Front Immunol. 2022 Aug 18;13:964919. doi: 10.3389/fimmu.2022.964919 (PMC9436409; doi:10.3389/fimmu.2022.964919)

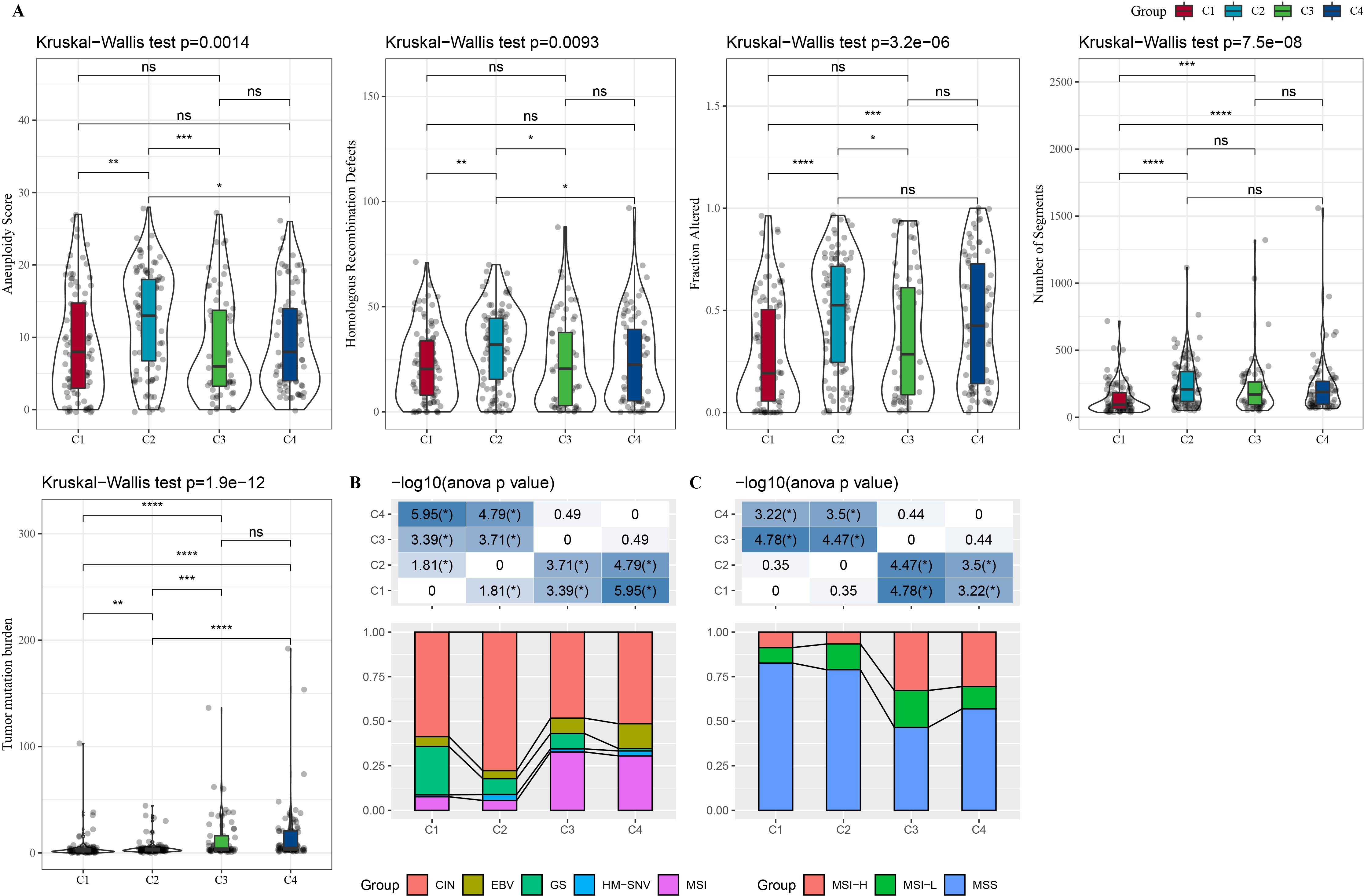

Supplement: Supplementary file 1 [file Image_1.jpeg]

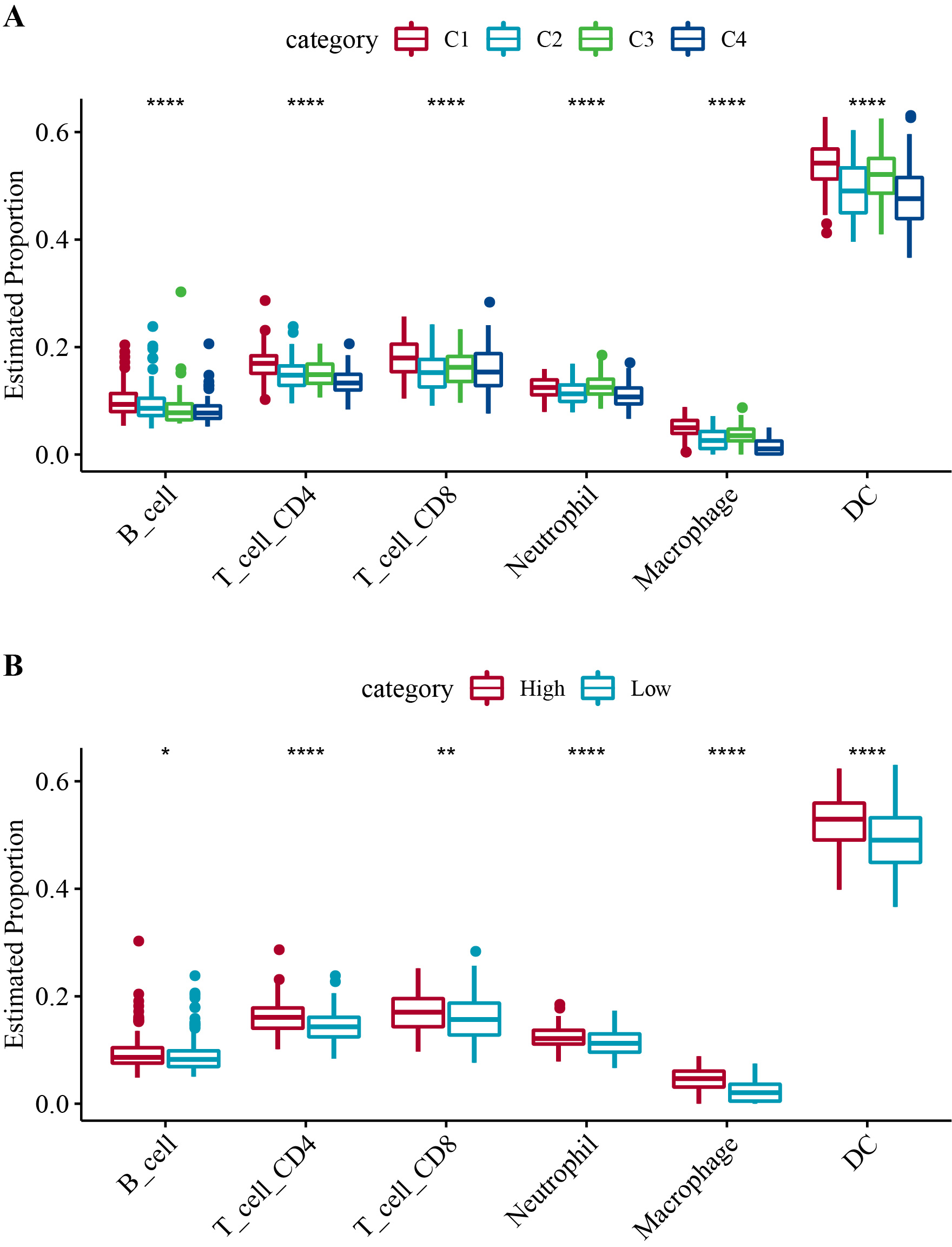

Supplement: Supplementary file 2 [file Image_2.jpeg]

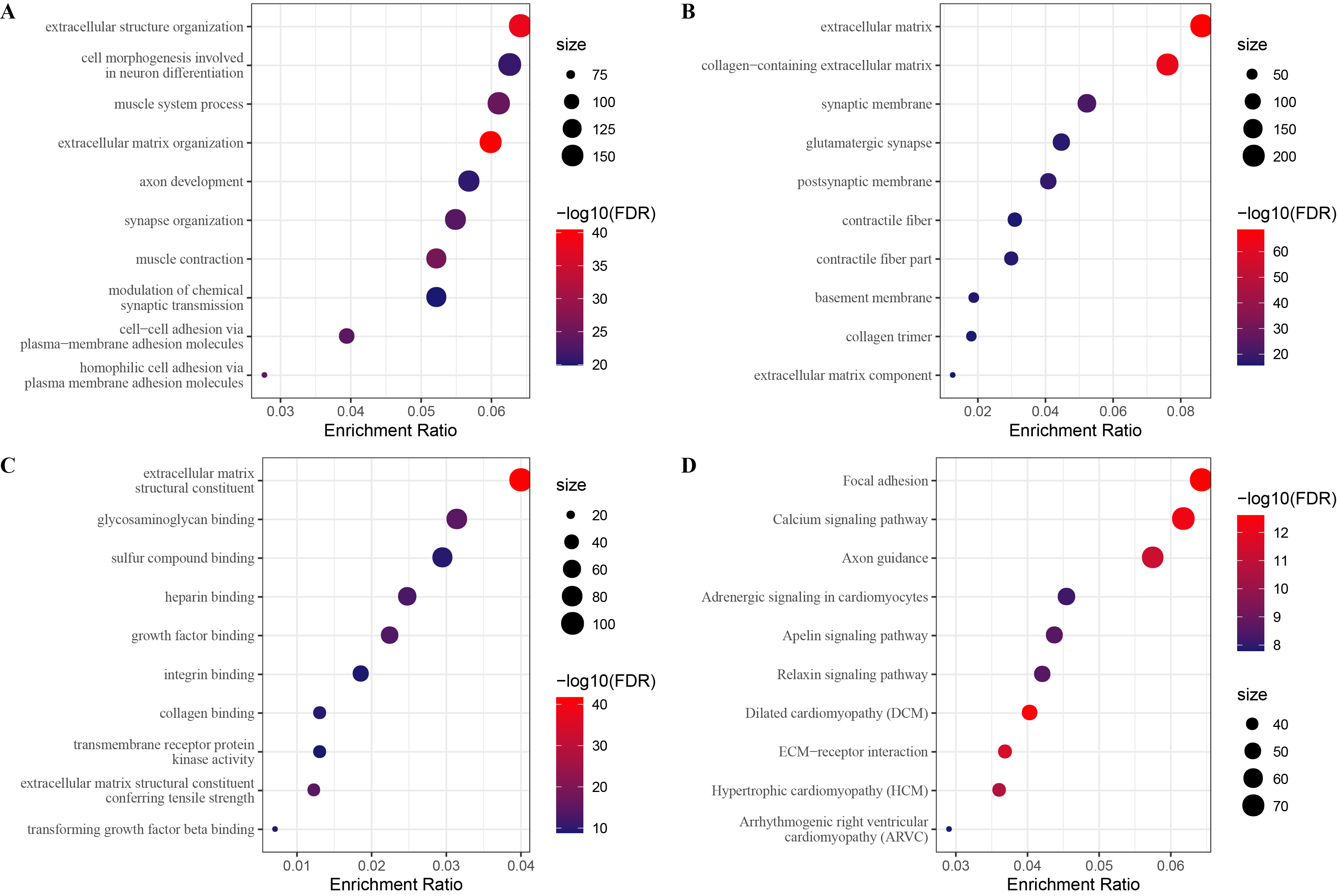

Supplement: Supplementary file 3 [file Image_3.jpeg]

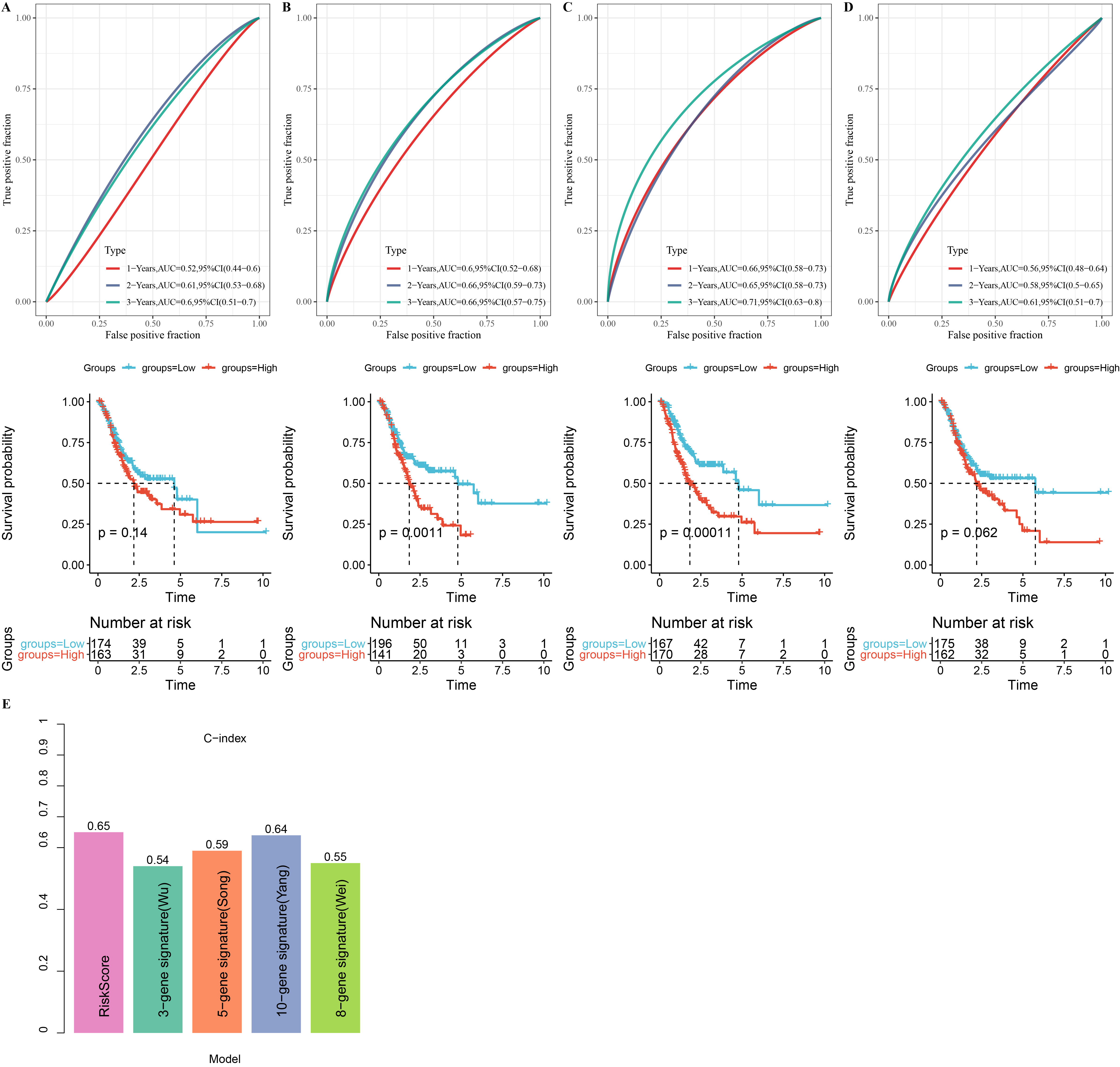

Supplement: Supplementary file 4 [file Image_4.jpeg]

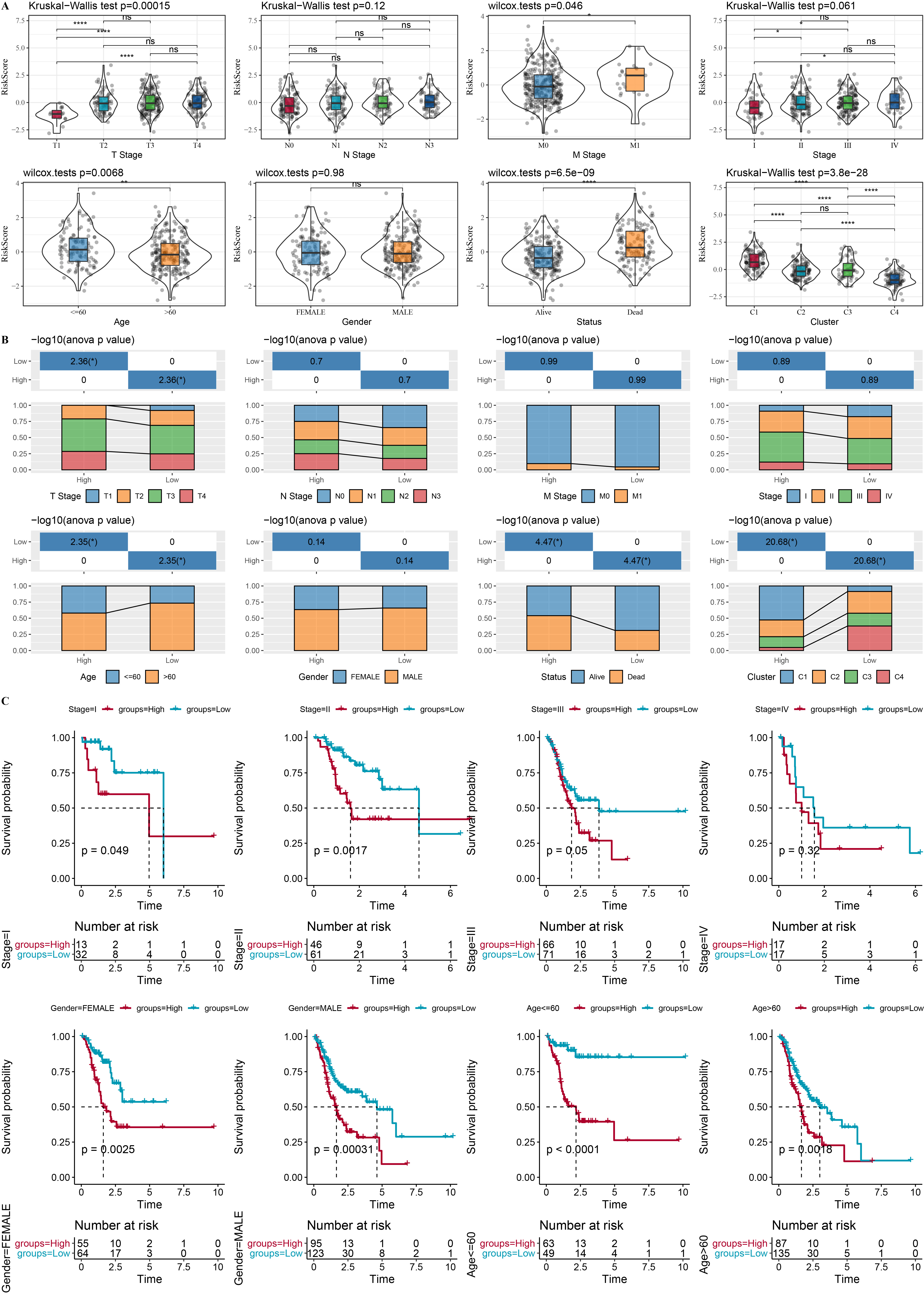

Supplement: Supplementary file 5 [file Image_5.jpeg]

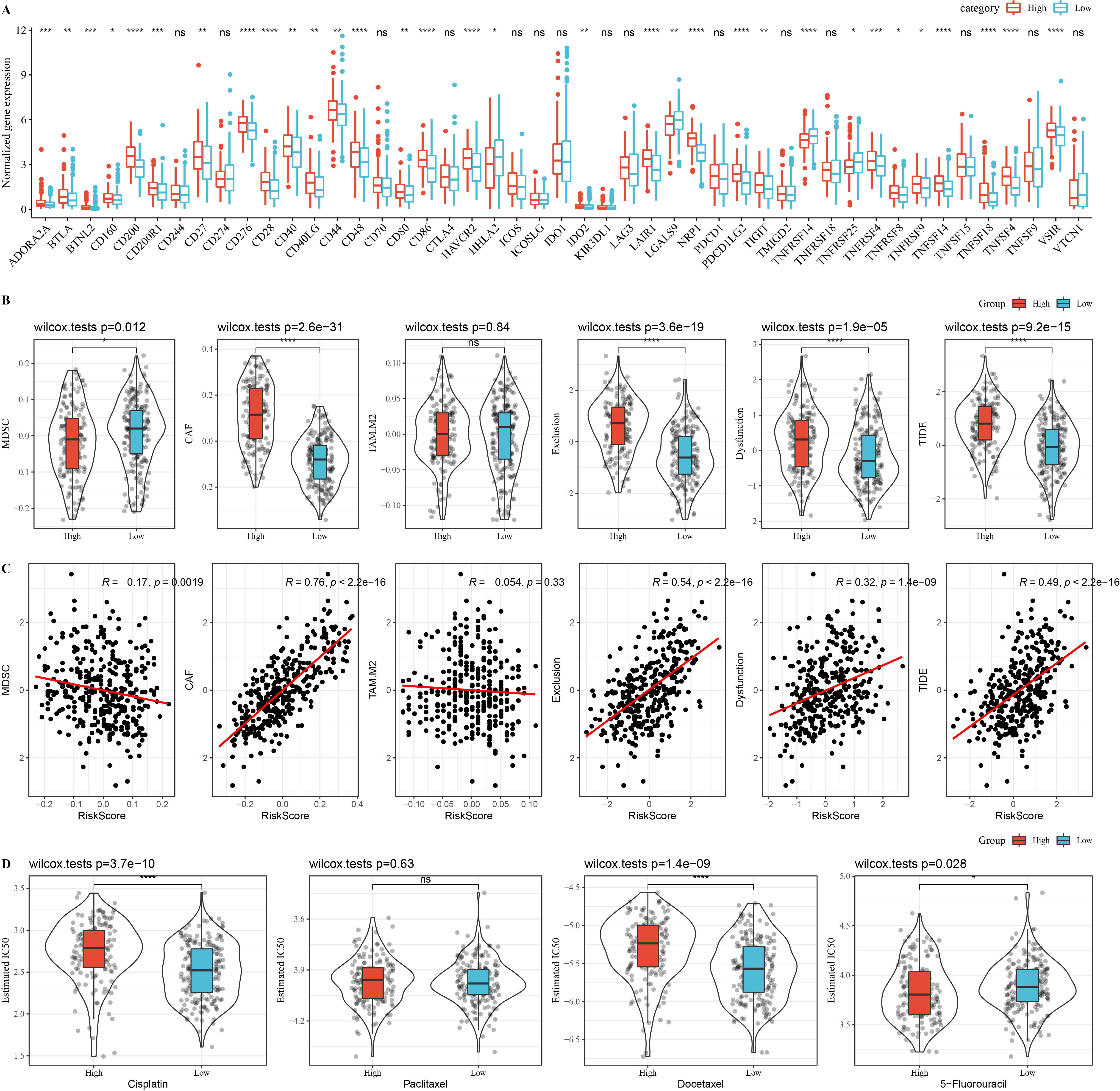

Supplement: Supplementary file 6 [file Image_6.jpeg]
